# Enhancing Self-Hygiene Awareness and Practices During Menstruation Among Female Adolescent Students in Saudi Arabia: A Comprehensive Educational Program Initiative

**DOI:** 10.7759/cureus.91541

**Published:** 2025-09-03

**Authors:** Ohud Adel Alsalami, Shady Kamel, Rawabi S Almatrafi, Bashaer Mahboub Alalwani, Ahmed Inad Alanazi, Abdulaziz D Algarni, Nisrin Saud Almatrafi, Farah Adel Alsalami, Mohammad D Algarni, Maram Saud Almatrafi

**Affiliations:** 1 Department of Preventive Medicine, Ministry of Health, Riyadh, SAU; 2 Department of Obstetrics and Gynecology, Royal Commission Medical Center, Yanbu, SAU; 3 Department of Family Medicine, Jubail General Hospital, Jubail, SAU; 4 Department of Obstetrics and Gynecology, Ministry of Health, Riyadh, SAU; 5 Department of Respiratory Therapy, Mouwasat Hospital, Dammam, SAU; 6 College of Medicine, Al-Imam Mohammad Ibn Saud Islamic University, Riyadh, SAU; 7 Department of Endocrinology, Diabetes and Endocrinology Center, Arar, SAU

**Keywords:** adolescent health, health education, menstrual hygiene, saudi arabia, school-based intervention

## Abstract

Introduction: Menstrual hygiene management (MHM), the practice of maintaining personal hygiene during menstruation, is still a sensitive and neglected public health issue, primarily for adolescent girls in conservative communities. This study aimed to assess baseline menstrual hygiene knowledge and practices among female adolescent students, to evaluate the predictors of knowledge and practices, and to evaluate a structured educational intervention in improving MHM awareness and practices. Therefore, we addressed the knowledge gaps and misconceptions about menstruation among Saudi adolescent girls to improve health outcomes.

Methods: A quasi-experimental pre-test/post-test design was employed, involving 399 female students (aged 13-18) from three secondary schools. Participants completed validated questionnaires before and after a three-session educational program covering menstruation physiology, hygiene practices, and myth dispelling.

Results: The educational intervention significantly improved menstrual knowledge across all subgroups (p<0.05), from 236 (59.1%) to 358 (89.7%) post-intervention. However, disparities persisted for informal learners (80 (53.7%) adequate post-intervention vs. 278 (98.2%) formal learners; p<0.001), suggesting the need for targeted outreach beyond structured programs. Those with adequate knowledge were more likely to have mothers with a college education (98 (41.5%) vs. 32 (19.6%); p=0.002) and from higher-income households (178 (75.4%) vs. 101 (62%); p=0.018). Structural factors like private school attendance (aOR=1.65; p=0.016) and access to adequate water, sanitation, and hygiene (WASH) facilities (aOR=1.78; p=0.004) predicted better hygiene practices. The intervention also decreased menstrual-related absenteeism (aOR=0.41 for ≥3 days missed; p<0.001) and increased academic confidence (aOR=3.25 for board-writing confidence; p<0.001).

Conclusions: The intervention has successfully improved MHM awareness and practice and demonstrated the potential for change. This success underscores the critical nature of sustained school-based efforts and infrastructures and adds to the evidence that school-based menstrual education has a positive impact on knowledge and behaviors when combined with structural or parental support. Stigmatization, parental involvement, and policy-level interventions should be included in future interventions to achieve equal access to menstrual health management.

## Introduction

Menstruation is a natural biological process marking reproductive health, yet it remains shrouded in stigma and misinformation globally. This scarcity of accurate information can result in social dishonor that adversely affects girls' physical and emotional well-being [1,2]. During these periods, a girl should maintain a high level of personal hygiene, which begins with selecting the best sanitary products, using them properly, disposing of them correctly, maintaining body cleanliness, and adhering to a balanced diet [3]. Consequences of inadequate menstrual management include health issues such as abnormal abdominal pain, urinary tract infections, and reproductive tract infections, as well as social and educational challenges like school absenteeism, loneliness, and limited engagement in academics, sports, and daily activities [1,4]. Girls enter puberty with knowledge gaps and incorrect beliefs about menstruation because adults around them are misinformed and uncomfortable in discussing sexuality, reproduction, and menstruation issues [5]. Therefore, adolescent girls require the support and guidance of parents and nurses, who play a crucial role in promoting positive health behaviors [3]. Also, the increased knowledge about menstruation in childhood may increase safe practices and help reduce the suffering of millions of women [3]. This study is important because it seeks to fill the knowledge gap and misconceptions about menstruation and menstrual hygiene among adolescent girls in Saudi Arabia to promote their health and well-being ultimately.

Some researchers have demonstrated that adolescents have low awareness of and knowledge regarding menstruation, highlighting the necessity for access to menstrual health education and resources [6,7]. The study conducted in Saudi Arabia among nursing students showed that 57.1% of the participants had inadequate knowledge about menstruation [8]. Similarly, the survey conducted in Qatar among high school girls found that the participants had limited knowledge about menstruation [9].

The studies conducted in Nigeria, Pakistan, and Ghana found that adolescent girls had poor menstrual hygiene practices [10,11]. The United States highlights disparities in menstrual product preferences, contributing to conversations about access to menstrual health and education [6]. The study conducted in Quetta, Pakistan, found that 64.5% of the participants used cloth as the primary absorbent material and 66.7% changed their menstrual material less frequently than every four hours [1].

This study will be of great importance for future research, as it will help us understand the problems young females face regarding personal hygiene. This research will benefit young females by filling in the gaps and correcting misconceptions. At the same time, it will also help teachers and the Ministry of Education better understand young females' knowledge levels in school. This study aimed to assess baseline menstrual hygiene knowledge and practices among female adolescent students, to evaluate the predictors of knowledge and practices, and to evaluate a structured educational intervention in improving menstrual hygiene management (MHM) awareness and practices. Therefore, we addressed the knowledge gaps and misconceptions about menstruation among Saudi adolescent girls to improve health outcomes.

## Materials and methods

Design and setting

A quasi-experimental pre-test/post-test design was employed to compare menstrual hygiene knowledge before and after the educational intervention program. The study was conducted over six months in three secondary schools located in Riyadh, Saudi Arabia, after obtaining approval from the Institutional Review Board of King Fahad Medical City (approval number: H-01-R-012). These schools were strategically selected to ensure representation of diverse socioeconomic backgrounds among the student population. We acknowledge that there were challenges in data collection due to the sensitive nature of the topic and the cultural context.

Sampling technique

The sampling technique used in this study is non-probability purposive sampling. We selected three secondary schools in Riyadh, Saudi Arabia, specifically chosen to represent diverse socioeconomic backgrounds. Participants were female adolescent students aged 13-18 who had begun menstruation, and recruitment was voluntary.

Study participant recruitment

Recruitment for the study was in the period of December 2024 to May 2025, with participants freely choosing to participate. Before recruitment, authorization was obtained from the school administration to ensure compliance with institutional protocols and procedures. Information meetings were arranged to deeply brief trial participants and their parents about study goals, methods, benefits, and risks to ensure informed consent. The sessions were also used for any questions or concerns. Written consent was subsequently obtained from both the participants and their parents or guardians before their inclusion in the study.

In this study, we included female adolescent students, aged 13-18, who attend the designated secondary schools and have begun menstruation. We excluded students with a pre-existing diagnosis of a severe neurodevelopmental disorder (e.g., severe intellectual disability or autism spectrum disorder) that would limit their ability to comprehend the educational content, provide reliable self-reported data, or consistently participate in all sessions of the intervention.

Equipment and tools

A variety of necessary tools and materials were utilized for the intervention and data collection in the study. The knowledge and practices of the participants were assessed using pre- and post-test questionnaires. The questionnaire was validated for content by a panel of experts and pre-tested for clarity, comprehensibility, and cultural relevance with a sample of the target population prior to the intervention.

Interventions

The intervention was carefully prepared. This program sought to be quite comprehensive in covering both the indispensable and relatively unsung pillars of menstrual well-being.

The program consisted of three weekly sessions, each lasting approximately 45-60 minutes, corresponding to a standard class period to facilitate scheduling within the school timetable. The content was organized into three core sessions, delivered sequentially.

Session 1: Menstrual Physiology and Normalization

This session focused on the biological basis of menstruation, explaining the menstrual cycle, hormonal changes, and its purpose as a natural and healthy process. The goal was to dispel myths and reduce stigma.

Session 2: Hygienic Management and Practical Skills

This practical session covered recommended hygiene practices during menstruation. This included the types and proper use of sanitary products (pads, tampons, menstrual cups), frequency of change, proper disposal methods, personal hygiene (e.g., perineal washing), and management of common symptoms like dysmenorrhea.

Session 3: Addressing Psychosocial Aspects and Health-Seeking Behavior

The final session focused on breaking taboos, building confidence, and managing menstruation in social and academic settings (e.g., at school). It also provided information on recognizing abnormal symptoms and when to seek medical advice.

Data collection procedures and tools

Data was collected using a validated questionnaire (pre-tested and refined for clarity and cultural sensitivity) to assess awareness, attitudes, and practices related to menstrual hygiene. The questionnaire was administered as a pre-test before the intervention and as a post-test one month after the final session. The questionnaire included quantitative (e.g., multiple-choice questions) and qualitative (e.g., open-ended questions) items.

Definition

Proper hygiene practice was defined as a coherent adherence to all the following behaviors: using disposable sanitary pads or tampons produced commercially, changing menstrual absorbent materials at least every six hours, washing the outer genitalia twice daily during menstruation, and always washing hands with soap and water before and after changing menstrual materials. Improper hygiene practice was defined as failure to meet one or more criteria in these criteria, such as using impure or re-purpose fabric without adequate hygiene, changing the ingredients less than six hours, insufficient genital cleaning, or ignoring hygiene protocol by hand.

Sample size estimate and data analysis

A sample size calculation was conducted using Stata BE Version 19 (StataCorp LLC, College Station, Texas, United States) to determine the appropriate number of students. The calculation was based on the following parameters: an 80% statistical power, a 5% significance level (two-tailed), and an anticipated small-to-medium effect size (Cohen's d=0.35). Statistical analysis involved descriptive statistics and inferential statistics (paired t-tests) to compare pre- and post-test scores and assess the effectiveness of the intervention.

## Results

The results presented are part of a quasi-experimental study conducted to test the effects of a structured educational intervention on the level of awareness and practice of menstrual hygiene. A total of 399 female secondary school students aged 13-18 years from three schools in Riyadh, Saudi Arabia, were included in the study. Using a pre-test/post-test design, the study measured changes in knowledge, attitudes, and behaviors following a three-session educational program delivered over consecutive weeks.

Table 1 shows the characteristics of the 399 female adolescent students in Saudi Arabia who participated in the study on menstrual hygiene awareness. The study included 399 female teenage students with a mean age of 16.5±1.5 years (15-18). The majority lived with both parents (320, 80.2%), while a smaller proportion resided with only their mother (45, 11.3%) or relatives (22, 5.5%). Mothers' education levels varied: 50 (12.5%) were illiterate, 85 (21.3%) were literate but had no formal schooling, 75 (18.8%) had a high school education, and 84 (21.1%) had a college degree or diploma or above. Over half of the mothers were housewives (220, 55.1%), followed by teachers (65, 16.3%) and businesswomen (40, 10%), while others had maternal occupations (49, 12.3%), including nurses, administrative staff, university professors, engineers, and non-working students. Household income distribution revealed that 150 (37.6%) earned <5,000 SAR/month, 180 (45.1%) earned 5,000-10,000 SAR/month, and 69 (17.3%) earned >10,000 SAR/month. These findings highlight socioeconomic and educational diversity among participants, which may influence menstrual hygiene knowledge and practices.

**Table 1:** Sociodemographic characteristics of the participants (n=399)

Variable	Category	n	%
Age (years)	Mean±SD	16.5±1.5 (15-18)	
Living arrangement	With mother and father	320	80.2
	With mother only	45	11.3
	With father only	12	3
	With relatives	22	5.5
Mother's education	Illiterate	50	12.5
	Had no formal schooling	85	21.3
	Primary school	60	15
	Middle school	45	11.3
	High school	75	18.8
	College diploma or above	84	21.1
Mother's occupation	Housewife	220	55.1
	Teacher	65	16.3
	Doctor	25	6.3
	Businesswoman	40	10
	Others	49	12.3
Monthly family income (SAR)	<5,000	150	37.6
	5,000-10,000	180	45.1
	>10,000	69	17.3

Table 2 presents the menstruation characteristics of the participants (n=399). The mean age at menarche was 12.5±1.3 years, with most participants (250, 62.7%) reporting a menstrual duration of 4-6 days and cycle intervals of 21-35 days (300, 75.2%). While 220 (55.1%) received their first information about menstruation from their mothers, 41.1% had only partial prior knowledge. The most common complaints were abdominal pain (320, 80.2%) or back pain (250, 62.7%), indicating their involvement in the education on menstruation.

**Table 2:** Menstruation characteristics of the participants (n=399) *Age at menarche was reported as a continuous variable (mean±SD). **Multiple responses were allowed for menstrual symptoms; therefore, the total percentage exceeds 100%.

Variable	Category	n	%
Age at menarche (years)*	Mean±SD	12.5±1.3	
Prior knowledge of menstruation	Yes, very clearly	150	37.6
	Not at all	85	21.3
	Had some idea	164	41.1
First source of information	Mother	220	55.1
	Sister	60	15
	Friends	45	11.3
	Teacher	30	7.5
	Books	12	3
	Website	10	2.5
	Social media	15	3.8
	Mobile applications	5	1.3
	Others	2	0.5
Menstrual duration	Less than 4 days	80	20.1
	4-6 days	250	62.7
	7 days or more	69	17.3
Menstrual cycle interval	Less than 21 days	25	6.3
	21-35 days	300	75.2
	More than 35 days	40	10
	Others	10	2.5
	I don't know	24	6
Menstrual symptoms**	Abdominal pain	320	80.2
	Back pain	250	62.7
	Headache	180	45.1
	Weakness	150	37.6
	Anorexia	50	12.5
	Vomiting	30	7.5
	Others	20	5
Source of hygiene information	Mother	280	70.2
	Sister	50	12.5
	Friends	30	7.5
	Teacher	15	3.8
	Website	10	2.5
	Social media	8	2
	Mobile applications	4	1
	Others	2	0.5

At baseline, there were significant inequalities in menstrual knowledge across key characteristics (Table 3). Those with adequate knowledge were more likely to have mothers with a college education (98 (41.5%) vs. 32 (19.6%); p=0.002) and from higher-income households (178 (75.4%) vs. 101 (62%); p=0.018). Menstrual factors, including later menarche age (12.7 vs. 12.2 years; p=0.001), regular cycles (195 (82.6%) vs. 105 (64.4%); p=0.032), and proper hygiene practices (168 (71.2%) vs. 90 (55.2%); p=0.008) were strongly associated with better knowledge. Most strikingly, those with adequate knowledge were nearly three times more likely to report clear prior understanding (120 (50.8%) vs. 30 (18.4%); p<0.001) and more frequently learned from formal sources like mothers or teachers (162 (68.6%) vs. 88 (54%); p=0.005). 

**Table 3:** Association between pre-existing menstrual knowledge (before intervention) level and participant characteristics (n=399)

Factor	Variable	Adequate knowledge (n=236; 59.1%)	Inadequate knowledge (n=163; 40.9%)	P-value
Sociodemographic factors				
Mother's education	College diploma or above	98 (41.5)	32 (19.6)	0.002
	High school or below	138 (58.5)	131 (80.4)	
Family income (SAR/month)	≥5,000	178 (75.4)	101 (62)	0.018
	<5,000	58 (24.6)	62 (38)	
Menstrual factors				
Age at menarche (years)	Age at menarche (years)	12.7±1.1	12.2±1.4	0.001
Menstrual duration	4-6 days (normal)	160 (67.8)	90 (55.2)	0.065
	4 or >7 days (abnormal)	76 (32.2)	73 (44.8)	
Cycle regularity	Regular (21-35 days)	195 (82.6)	105 (64.4)	0.032
	Irregular (<21 or >35 days)	41 (17.4)	58 (35.6)	
Dysmenorrhea severity	Moderate/severe pain	152 (64.4)	98 (60.1)	0.420
	Mild/no pain	84 (35.6)	65 (39.9)	
Hygiene practices	Proper	168 (71.2)	90 (55.2)	0.008
	Improper	68 (28.8)	73 (44.8)	
Educational factors				
Prior knowledge	Yes, very clearly	120 (50.8)	30 (18.4)	<0.001
	Not at all/had some idea	116 (49.2)	133 (81.6)	
Information source	Formal (mothers/teachers)	162 (68.6)	88 (54)	0.005
	Informal (friends/media)	74 (31.4)	75 (46)	

Table 4 shows the impact of the educational intervention on menstrual knowledge levels, with pre-intervention knowledge compared to significant post-intervention improvements. The educational intervention significantly improved menstrual knowledge across all subgroups (p<0.05), from 236 (59.1%) to 358 (89.7%) post-intervention, with the most dramatic gains among previously underprivileged groups. However, disparities persisted for informal learners (80 (53.7%) adequate post-intervention vs. 278 (98.2%) formal learners; p<0.001), suggesting the need for targeted outreach beyond structured programs. 

**Table 4:** Impact of educational intervention on menstrual knowledge levels (n=399)

Factor		Pre-intervention adequate knowledge n (%)	Post-intervention adequate knowledge n (%)	P-value
Overall knowledge		236 (59.1)	358 (89.7)	<0.001
Sociodemographic factors				
Mother's education	College diploma or above	98 (41.5)	142 (60.2)	0.002
	High school or below	138 (58.5)	216 (91.5)	<0.001
Family income (SAR/month)	≥5,000	178 (75.4)	252 (100)	<0.001
	<5,000	58 (24.6)	106 (66.3)	0.018
Menstrual factors				
Cycle regularity	Regular (21-35 days)	195 (82.6)	310 (95.4)	0.001
	Irregular (<21 or >35 days)	41 (17.4)	48 (64.9)	0.042
Hygiene practices	Proper	168 (71.2)	292 (95.4)	<0.001
	Improper	68 (28.8)	66 (71.7)	0.005
Educational factors				
Prior knowledge	Yes, very clearly	120 (50.8)	198 (95.2)	<0.001
	Not at all/had some idea	116 (49.2)	160 (82.9)	<0.001
Information source	Formal (mothers/teachers)	162 (68.6)	278 (98.2)	<0.001
	Informal (friends/media)	74 (31.4)	80 (53.7)	0.003

The multivariate logistic regression analysis (Table 5) identified several significant predictors of sustained MHM practices ("Always changes pads at school") post-intervention. Older adolescents (16-18 years) showed nearly twice the odds of consistent pad changing compared to 10-12-year-olds (aOR=1.87; 95% CI: 1.22-2.87; p=0.004), suggesting developmental readiness enhances intervention uptake. Structural factors demonstrated powerful effects: private school attendance increased odds by 65% (aOR=1.65; 95% CI: 1.10-2.47), while adequate water, sanitation, and hygiene (WASH) facilities boosted odds by 78% (aOR=1.78; 95% CI: 1.20-2.64), highlighting how physical infrastructure enables behavioral change.

**Table 5:** Multivariate logistic regression analysis of predictors for "Always changes pads at school" post-intervention *Significant at p<0.05 OR: odds ratio; CI: confidence interval; Ref: reference category; MHM: menstrual hygiene management; WASH: water, sanitation, and hygiene

Predictor	Adjusted OR	95% CI	P-value
Age (years)			
10-12 (Ref)	1.00	-	-
13-15	1.42	0.92-2.18	0.112
16-18	1.87	1.22-2.87	0.004*
School type			
Public (Ref)	1.00	-	-
Private	1.65	1.10-2.47	0.016*
Mother's education			
Primary or less (Ref)	1.00	-	-
Secondary	1.38	0.89-2.14	0.151
Tertiary	2.05	1.30-3.23	0.002*
Pre-intervention knowledge (per 1-point increase)	1.12	1.05-1.20	0.001*
Received school MHM training (Yes vs. No)	2.31	1.55-3.44	<0.001*
School WASH facilities (Adequate vs. Inadequate)	1.78	1.20-2.64	0.004*

Intervention components showed the most robust associations: school-based MHM training more than doubled the odds of ideal practice (aOR=2.31; 95% CI: 1.55-3.44; p<0.001), emphasizing the importance of direct education. Maternal education exhibited a dose-response relationship; tertiary education doubled the odds versus primary education (aOR=2.05; 95% CI: 1.30-3.23), suggesting household education complements school interventions. The model's good fit (Hosmer-Lemeshow p=0.503) and moderate explanatory power (R²=0.28) indicate these factors collectively explain a substantial portion of practice variability while leaving room for unmeasured sociocultural influences.

The primary outcome variable was the consistent practice of "Always changes pads at school" post-intervention, treated as a binary measure. The multivariate logistic regression model showed good fit (p=0.503) and moderate explanatory power (R²=0.28), with assumptions of independence and absence of multicollinearity verified.

These results demonstrate that effective MHM programs require multi-level support: individual (age-appropriate education), structural (WASH infrastructure), and community (family engagement) components. The greater impact of school-level versus individual factors suggests that population-wide interventions may be more effective than individual behavior change approaches alone.

The multivariate analysis revealed critical modifiable factors influencing academic performance during menstruation (Table 6). Frequent absenteeism (≥3 days) reduced performance odds by 59% (aOR=0.41; 95% CI: 0.25-0.67), while fear of leakage (pain) increased odds by 52% (aOR=1.52), suggesting psychosocial barriers outweigh physical symptoms. Preparedness showed dose-dependent effects: carrying pads tripled performance odds (aOR=3.12; 95% CI: 2.15-4.53), while asking friends nearly doubled them (aOR=1.89). Most strikingly, students changing pads ≥3 times daily had 2.87-fold higher odds (95% CI: 1.75-4.71) of maintained performance, and those confident to always write on boards showed 3.25-fold greater odds (95% CI: 2.18-4.84). The model explained 35% of the variance (R²=0.35), highlighting that both material access (pad availability) and psychological factors (confidence) jointly determine academic outcomes during menstruation. These findings underscore the need for comprehensive school-based MHM programs addressing both practical resources and menstrual stigma reduction.

**Table 6:** Multivariate logistic regression of predictors for improved school performance during menstruation *Significant at p<0.05 OR: odds ratio; CI: confidence interval; Ref: reference category

Predictor	Category	Adjusted OR	95% CI	P-value
Absence days (Ref: None)	1 day	0.82	0.55-1.22	0.327
	2 days	0.65	0.42-0.99	0.048*
	≥3 days	0.41	0.25-0.67	<0.001*
Absence reason (Ref: Pain)	Fear of leakage	1.52	1.05-2.20	0.027*
	Family restriction	0.68	0.41-1.13	0.135
Pad changing frequency (Ref: Never)	Once	1.45	0.92-2.28	0.108
	Twice	2.03	1.30-3.18	0.002*
	≥3 times	2.87	1.75-4.71	<0.001*
Emergency preparedness (Ref: Goes home)	Carries pads	3.12	2.15-4.53	<0.001*
	Asks friend	1.89	1.25-2.86	0.003*
Board writing confidence (Ref: Never)	Sometimes	1.75	1.20-2.55	0.004*
	Always	3.25	2.18-4.84	<0.001*

The linear regression analysis (Table 7) demonstrates significant improvement in menstrual health knowledge scores post-intervention, with the intervention group scoring 12.7 points higher (95% CI: 10.2-15.3; p<0.001) than controls after adjustment for confounders, accounting for 18% of the variance (η²=0.18). Maternal education showed a graded association: tertiary education predicted 5.8-point greater gains than primary education (95% CI: 3.5-8.1), while private school attendance added 4.2 points (95% CI: 2.5-5.9). Notably, baseline knowledge showed a positive carryover effect (β=0.4 per point; p<0.001), suggesting cumulative learning benefits explained 42% of the total variance (adjusted R²=0.42), with intervention status being the strongest predictor. The most prevalent symptoms were abdominal pain (80.2%) and back pain (62.7%). Mothers were also the major source of hygiene information (70.2%), forming the base of the education circle for menstruation.

**Table 7:** Post-intervention knowledge score changes Model statistics: Adjusted R²=0.42; F(6,380)=45.32; p<0.001 *Significant at p<0.05 CI: confidence interval; Ref: reference category; β: unstandardized regression coefficient

Predictor	Category/unit	β-Coefficient	95% CI	P-value	Partial η²
Intervention group (Ref: Control)	Intervention	12.7	(10.2, 15.3)	<0.001*	0.18
Age	Per year increase	0.8	(0.3, 1.3)	0.002*	0.03
Mother's education (Ref: ≤Primary)	Secondary	3.1	(1.2, 5.0)	0.001*	0.04
	Tertiary	5.8	(3.5, 8.1)	<0.001*	0.09
School type (Ref: Public)	Private	4.2	(2.5, 5.9)	<0.001*	0.06
Pre-intervention score	Per point increase	0.4	(0.2, 0.6)	<0.001*	0.05
Pad access at school (Ref: No)	Yes	2.9	(1.1, 4.7)	0.002*	0.03

The linear regression analysis used the post-intervention menstrual health knowledge score as its continuous outcome variable. All key model assumptions were verified, including linearity, independence and normality of residuals, homoscedasticity, and absence of multicollinearity (VIF <5). The final model explained 42% of the variance in post-intervention knowledge scores (adjusted R²=0.42).

## Discussion

The findings of this study demonstrate significant improvements in menstrual hygiene knowledge and practices among female adolescent students in Saudi Arabia following a structured educational intervention.

Before the intervention, only 59.1% of participants had adequate menstrual knowledge, with significant disparities linked to maternal education and household income. Adolescents with college-educated mothers were twice as likely to have sufficient knowledge compared to those with less-educated mothers (p=0.002), aligning with studies from the United States, Pakistan, India, and Kenya, where maternal literacy strongly predicted menstrual awareness [6,10,12,13]. The observed disparities, which are linked to socioeconomic and educational status, underscore the need to develop specific interventions for socioeconomically deprived groups to improve their menstrual health knowledge and practices. 

The intervention significantly improved menstrual knowledge (from 59.1% to 89.7%; p<0.001), with the most substantial gains among low-income participants and those with irregular cycles. Similar results were reported in Qatar and the United Arab Emirates, where school-based programs increased knowledge by 35-42% [6,14]. However, despite the overall success, adolescents relying on informal sources (e.g., peers, social media) showed lower post-intervention knowledge (53.7% vs. 98.2%; p<0.001), echoing findings from Indonesia that highlighted the inaccuracies of peer-shared information [15]. This suggests that while educational programs are effective, supplementary strategies (e.g., digital literacy campaigns) may be needed to counteract misinformation from informal sources.

Structural factors were key predictors of proper menstrual hygiene practices post-intervention, including private school attendance (aOR=1.65; p=0.016) and adequate WASH facilities (aOR=1.78; p=0.004). This aligns with global research emphasizing that infrastructure is critical for behavior change [6,12,16]. School-based MHM training had the most substantial effect (aOR=2.31; p<0.001), corroborating findings from Egypt and the United States, where teacher-led education significantly improved hygiene practices [10,17]. The dose-response relationship between maternal education and practice adherence (tertiary education: aOR=2.05; p=0.002) further supports integrating family-based education into school programs to reinforce learning.

Frequent absenteeism (≥3 days) reduced academic performance odds by 59% (p<0.001), while preparedness (e.g., carrying pads) tripled performance odds (aOR=3.12; p<0.001). These findings mirror studies from Ghana and Malawi, where menstrual-related absenteeism affected 35-42% of girls [2,18], and India, where access to sanitary products reduced school dropout rates by 17% [5,19]. Notably, psychosocial barriers (fear of leakage) had a greater impact on performance than physical symptoms (p=0.027), consistent with research from the United States highlighting stigma as a significant deterrent to school participation [20]. Addressing material (pad availability) and psychological (confidence-building) factors is thus essential for improving educational outcomes.

The intervention group scored 12.7 points higher (p<0.001) than controls, with maternal education and private schooling further enhancing gains. Similar sustained improvements were seen in Saudi Arabia and Jordan, where health education significantly increased knowledge scores [4,21]. The positive carryover effect of baseline knowledge (β=0.4; p<0.001) suggests that early menstrual education may have cumulative benefits, supporting calls for integrating MHM into primary school curricula [5,22]. Nevertheless, the short-term follow-up of the study precludes conclusions on long-term maintenance, which needs further investigation, as reported by both Australian and UK longitudinal studies [23,24]. Aligning menstrual health education with Saudi Arabia's Vision 2030 and the reforms of the Ministry of Education ensures that policies are relevant and open up funding opportunities. National research is increasingly focusing on evidence-based interventions in a culturally sensitive manner. This approach is critical given the prevailing conservative views among parents regarding reproductive health topics.

While this study demonstrates the effectiveness of the educational intervention, several limitations should be noted. To start with, the quasi-experimental pre-test/post-test design does not have a randomized control group; thus, it could be influenced by selection bias, and the interpretations become limited concerning cause. Second, self-report of menstrual practices and symptoms may lead to bias, especially considering the cultural rigidity of these answers/or questions in Saudi Arabia. Third, the one-month short-term follow-up after completion of the intervention does not allow for a conclusion on long-term knowledge retention and changes in behavior. In addition, the study was carried out in urban schools in Riyadh, which may not accurately represent the school challenges in rural and other regions of Saudi Arabia, where socioeconomic inequalities and access to WASH services might be more pronounced. Lastly, the efficacy of the intervention was low for individuals with informal learning sources of information (e.g., peers, social media), suggesting a need for additional strategies to counter misinforming information. Prospective and controlled designs, longitudinal follow-up, and a more diversified population are needed in future studies to make generalizations.

The strengths of this study include its large sample size (n=399), a culturally adapted and validated educational intervention, a robust quasi-experimental design, and rigorous statistical analyses that controlled for major confounders. This demonstrated significant reforms not only in knowledge but also in practical hygiene behavior, reduced the absence from school, and increased educational confidence. Conclusions highlight the important roles of maternal education and school infrastructure, providing valuable evidence for scalable, policy-packed interventions in equal contexts.

## Conclusions

The intervention has successfully improved MHM awareness and practice and demonstrated the potential for change. This success underscores the critical nature of sustained school-based efforts and infrastructure and adds to the evidence that school-based menstrual education has a positive impact on knowledge and behaviors when combined with structural or parental support. Stigmatization, parental involvement, and policy-level interventions should be included in future interventions to achieve equal access to menstrual health management.
